# Effectiveness of a combination strategy for linkage and retention in adult HIV care in Swaziland: The Link4Health cluster randomized trial

**DOI:** 10.1371/journal.pmed.1002420

**Published:** 2017-11-07

**Authors:** Margaret L. McNairy, Matthew R. Lamb, Averie B. Gachuhi, Harriet Nuwagaba-Biribonwoha, Sean Burke, Sikhathele Mazibuko, Velephi Okello, Peter Ehrenkranz, Ruben Sahabo, Wafaa M. El-Sadr

**Affiliations:** 1 ICAP at Columbia University, New York, New York, United States of America; 2 Department of Medicine, Weill Cornell Medical College, New York, New York, United States of America; 3 Department of Epidemiology, Mailman School of Public Health, Columbia University, New York, New York, United States of America; 4 Ministry of Health, Kingdom of Swaziland, Mbabane, Swaziland; 5 Bill and Melinda Gates Foundation, Seattle, Washington, United States of America; San Francisco General Hospital, UNITED STATES

## Abstract

**Background:**

Gaps in the HIV care continuum contribute to poor health outcomes and increase HIV transmission. A combination of interventions targeting multiple steps in the continuum is needed to achieve the full beneficial impact of HIV treatment.

**Methods and findings:**

Link4Health, a cluster-randomized controlled trial, evaluated the effectiveness of a combination intervention strategy (CIS) versus the standard of care (SOC) on the primary outcome of linkage to care within 1 month plus retention in care at 12 months after HIV-positive testing. Ten clusters of HIV clinics in Swaziland were randomized 1:1 to CIS versus SOC. The CIS included point-of-care CD4+ testing at the time of an HIV-positive test, accelerated antiretroviral therapy (ART) initiation for treatment-eligible participants, mobile phone appointment reminders, health educational packages, and noncash financial incentives. Secondary outcomes included each component of the primary outcome, mean time to linkage, assessment for ART eligibility, ART initiation and time to ART initiation, viral suppression defined as HIV-1 RNA < 1,000 copies/mL at 12 months after HIV testing among patients on ART ≥6 months, and loss to follow-up and death at 12 months after HIV testing. A total of 2,197 adults aged ≥18 years, newly tested HIV positive, were enrolled from 19 August 2013 to 21 November 2014 (1,096 CIS arm; 1,101 SOC arm) and followed for 12 months. The median participant age was 31 years (IQR 26–39), and 59% were women. In an intention-to-treat analysis, 64% (705/1,096) of participants at the CIS sites achieved the primary outcome versus 43% (477/1,101) at the SOC sites (adjusted relative risk [RR] 1.52, 95% CI 1.19–1.96, *p* = 0.002). Participants in the CIS arm versus the SOC arm had the following secondary outcomes: linkage to care regardless of retention at 12 months (RR 1.08, 95% CI 0.97–1.21, *p* = 0.13), mean time to linkage (2.5 days versus 7.5 days, *p* = 0.189), retention in care at 12 months regardless of time to linkage (RR 1.48, 95% CI 1.18–1.86, *p* = 0.002), assessment for ART eligibility (RR 1.20, 95% CI 1.07–1.34, *p* = 0.004), ART initiation (RR 1.16, 95% CI 0.96–1.40, *p* = 0.12), mean time to ART initiation from time of HIV testing (7 days versus 14 days, *p* < 0.001), viral suppression among those on ART for ≥6 months (RR 0.97, 95% CI 0.88–1.07, *p* = 0.55), loss to follow-up at 12 months after HIV testing (RR 0.56, 95% CI 0.40–0.79, *p* = 0.002), and death (*N* = 78) within 12 months of HIV testing (RR 0.80, 95% CI 0.46–1.35, *p* = 0.41). Limitations of this study include a small number of clusters and the inability to evaluate the incremental effectiveness of individual components of the combination strategy.

**Conclusions:**

A combination strategy inclusive of 5 evidence-based interventions aimed at multiple steps in the HIV care continuum was associated with significant increase in linkage to care plus 12-month retention. This strategy offers promise of enhanced outcomes for HIV-positive patients.

**Trial registration:**

ClinicalTrials.gov NCT01904994.

## Abstract 2

In a cluster-randomized trial done in Swaziland, Margaret McNairy and colleagues test a combined intervention for linking and retaining adults with HIV infection in care.

## Author summary

**Why was this study done?:**

- Linkage to care, retention in care, and achievement of viral load suppression on antiretroviral therapy (ART) among HIV-positive adults are necessary in order to achieve optimal health outcomes in terms of reduced morbidity and mortality and to decrease the risk of HIV transmission to others.
- Barriers to linkage to and retention in care are multifactorial and include both individual- and health system-level factors.
- We hypothesized that a multicomponent strategy using a combination of evidence-based interventions was needed to address the multiple gaps in linkage to and retention in care across the HIV care continuum.

**What did the researchers do and find?:**

- Ten clusters of affiliated HIV clinics in Swaziland were randomized to receive the standard of care (SOC; 1,101 participants) or a combination intervention strategy (CIS; 1,096 participants). The CIS included provision of participants with point-of-care CD4+ count testing at time of HIV testing, accelerated ART initiation among eligible patients, mobile phone appointment reminders, health educational packages, and noncash financial incentives.
- Participants were followed for 12 months from the time of testing HIV positive, and the primary study outcome was prompt linkage to care within 1 month of testing HIV-positive plus retention in care at 12 months after testing HIV positive. Secondary outcomes included additional steps in the HIV care continuum.
- We found that participants receiving care at HIV clinics randomized to the CIS study arm, as compared to the SOC study arm, were significantly more likely to achieve the primary outcome of prompt linkage to care plus 12-month retention (64% in the CIS arm versus 43% in the SOC arm, relative risk [RR] 1.52, 95% CI 1.19–1.96, *p* = 0.002).
- We also found that participants at CIS sites versus SOC sites had faster linkage to care, were more likely to be assessed for ART initiation, and had faster time to ART start. However, we did not find significant differences in viral suppression or mortality at 12-months after testing HIV positive.

**What do these findings mean?:**

- The Link4Health study showed that a CIS was 50% more effective than the SOC on prompt linkage to HIV care plus 12-month retention after HIV-testing and that the effect appeared to be primarily driven by enhanced retention in care.
- Limitations of this study include a small number of clusters and the inability to evaluate the contribution of each of the components of the strategy to the effect noted.
- The combination strategy used in this study could be easily adapted to other resource-limited settings and may be relevant to the challenges faced in engaging HIV-positive vulnerable and key populations.

## Introduction

Achieving the desired impact of HIV treatment, as measured by individual health outcomes and reduced transmission to others, is contingent upon completing all steps in the HIV care continuum, from identifying all individuals who are living with HIV and linking those found to be HIV positive to HIV care to retaining them in lifelong care and on antiretroviral therapy (ART) [1]. Over the past decade, the scale-up of HIV programs has been substantial, with over 18 million persons having initiated ART by the end of 2015 in low- and middle-income countries and an associated substantial decrease in HIV-related morbidity and mortality, as well as evidence of a decrease in HIV incidence in many of the most severely affected countries [2]. However, in order to achieve epidemic control, further optimization of the HIV care continuum is needed so as to achieve the Joint United Nations Programme on HIV/AIDS (UNAIDS) 90/90/90 targets, which require that 90% of individuals with HIV are aware of their diagnosis, that 90% of those aware of their HIV infection are initiated on ART, and that 90% of those on treatment achieve and maintain viral suppression [3].

Findings from HIV programs indicate that linkage to and retention in HIV care currently fall far short of the desired goals [4–6]. Linkage of HIV-positive individuals to HIV care varies from less than half of individuals linking to care within 6 months of an HIV-positive test to 72% who ever linked [5,7,8]. Once linked to care, less than half of HIV-positive patients are retained in care prior to initiation of ART, with only two-thirds of ART-eligible individuals initiating ART [5,7]. Lastly, only approximately three-quarters of patients initiated on ART have been noted to be retained in care at 12 months, with retention decreasing over the ensuing follow-up years [4].

Barriers to linkage to and retention in care are multifactorial and include both individual- and health system-level factors such as stigma, fear of disclosure, distance and cost of travel to clinic, attitudes of providers, and cumbersome clinic procedures with long waiting times [9,10]. Previous studies that aimed to overcome such barriers have largely focused on the assessment of 1 intervention primarily targeting a single step in the HIV care continuum [11–14]. We postulated that in order to address the multiple gaps across the care continuum, a multi-component intervention strategy is needed, with each component targeting one or more steps in the HIV care continuum.

The Link4Health study evaluated the effectiveness of a combination intervention strategy (CIS) utilizing 5 evidence-based interventions that address structural, behavioral, and biomedical barriers across the continuum of care, to improve linkage to and retention in care among newly identified HIV-positive adults in Swaziland.

## Methods

### Ethics

The study was approved by the institutional review boards at Columbia University and the Swaziland Scientific and Ethics Committee.

### Study design

A detailed description of the study methods was previously reported [15]. In brief, Link4Health was an implementation science study using a cluster site-randomized trial design. The study unit of randomization consisted of a public secondary-level HIV clinic paired with its largest affiliated public primary-level HIV clinic. Ten study units were selected from a total of 11 existing secondary-level HIV clinics in the country, based on clinic patient volume. Study units were pair matched, first by implementing partner (matching the 2 study units from implementing partner A) and then by location (urban [4] versus rural [4]) and clinic size, based on the estimated number of adults testing HIV positive in the 2 years prior to study implementation (<50 versus >50 per month for rural study units and <100 versus >100 per month for urban study units). Matched study units were randomized by a computerized random number generator to the CIS or standard of care (SOC) study arm. A cluster design was chosen to avoid disruption of service delivery, enable better fit within the routine workings at the clinical site, and allow the clinic staff to more easily implement the study. The study staff and clinic providers at each study unit were not blinded to the assigned arm for the site.

### Study setting and population

Swaziland is located in Southern Africa and has the world’s highest HIV prevalence, with HIV as the leading cause of death in the country [16]. The estimated adult (age 18–49 years) HIV prevalence is 31%, and the estimated incidence is 2.4% (95% CI 2.1–2.8) [17, 18]. The country has made impressive strides in responding to the epidemic, with nearly 70% of persons estimated to be living with HIV having initiated ART as of 2015 [19]. Nevertheless, historic rates of linkage to and retention in care at 12 months after ART initiation remain suboptimal [20]. Data available from Swaziland at the time of the initiation of the study showed that among 1,105 adults who tested HIV positive at community testing venues, only 37% linked to HIV care within 12 months of HIV testing [21]. Retention among adults at 36 months after ART start was 68% in 2011 per national estimates [22].

Inclusion criteria were as follows: adults aged ≥18 years, newly tested HIV positive, and willing to receive HIV care at the study unit and consent to study procedures. Exclusion criteria were as follows: planning on leaving the community during the study, prior enrollment in HIV care or initiation of ART in the past 6 months, currently on ART, reports a current pregnancy, or not able to speak English or SiSwati.

### Study interventions

All adults who tested HIV positive at participating sites were informed of the study by their providers. Interested individuals were referred to a study nurse who provided further information, confirmed eligibility, and, if eligible, obtained written consent. All consenting participants provided baseline information and thereafter were managed based on the study arm to which the clinic was randomly assigned.

### SOC

Participants at study units randomized to the SOC arm were managed according to country guidelines. These guidelines recommend that individuals identified as HIV positive receive post-test counseling and be referred to an HIV clinic using a national referral form [21]. Thereafter, upon presentation at their first HIV clinic visit, such individuals are to present their referral form, receive a clinical assessment, and have blood drawn for a CD4+ count test as well as hematology and chemistry tests and are instructed to return in 1–2 weeks for receipt of their results. Upon return, those eligible for ART according to then prevailing national guidelines (i.e., with a CD4+ count ≤ 350 cells/mm3) are to receive the first of 3 counseling sessions. Patients who are prescribed ART are instructed to return to the clinic every month for 6 months and then every 3 months, if they are stable on treatment. Patients who are ineligible for ART are instructed to return to clinic every 3 months for follow-up. Peer counselors are encouraged to call patients within 7 days of a missed clinic appointment. All patients are prescribed cotrimoxazole prophylaxis, and condoms, and health informational materials are to be made available in the clinics.

### CIS

Participants at clinics randomized to the CIS arm received a multicomponent strategy of 5 evidence-based interventions, targeting structural, biomedical, and behavioral barriers, which are described briefly below (Table 1) [15]. All components of the combination strategy utilized in this study were selected based on evidence of their effectiveness, feasibility, and suitability to patients in diverse healthcare settings.

**Table 1:** Comparison of combination intervention strategy (CIS) to standard of care (SOC) procedures.

Intervention	Standard of care (SOC)	Combination intervention strategy (CIS)	Type of intervention	Step targeted in HIV care continuum
**Point-of-care CD4+ count testing**	• Point-of-care CD4 assays available in some primary care clinics and some secondary health centers/hospitals for patients enrolled in HIV care but not at the HIV testing site • CD4+ count (Cyflow and FACS Caliber) after linkage to HIV care in the clinic or lab • Turnaround time approximately 2 weeks	• Point-of-care CD4 assays at the HIV testing site at the time of HIV testing • Turnaround time immediate	Structural and biomedical	Linkage, ART eligibility assessment, and ART initiation
**Accelerated ART initiation**	ART initiation per national guidelines for patients with CD4+ count ≤ 350 cells/mm^3^ or WHO Stage III/VI • Requires 3 counseling sessions and receipt of baseline lab tests • Initiation 2 weeks to 1 month from determination of ART eligibility	• Accelerated ART initiation for patients with point-of-care CD4+ count ≤ 350 cells/mm^3^ within 1 week from testing • Two counseling sessions (1 at the time of HIV testing and the other at the first HIV clinic visit) and collection of blood for other baseline lab tests • Initiation of ART for eligible patients prior to return of results with use of a checklist	Structural and biomedical	ART initiation and retention
**Cellular phone visit reminders**	• Telephone call within 7 days of missed visit for ART patients only	• SMS (or voice if illiterate) visit reminders 3 days prior to each scheduled visit • SMS (or voice if illiterate) reminder within 7 days after a missed visit for all patients	Behavioral	Linkage and retention
**Health education packages**	• Cotrimoxazole was prescribed for all patients once enrolled in HIV care • Condoms available	• A health education package was provided approximately every 3 months at visits. Packages included condoms, soap, cotrimoxazole, a pill box, and pictorial education about use of materials and HIV	Biomedical and behavioral	Retention
**Noncash financial incentive**	• None	• Noncash financial incentive (mobile airtime) were provided for those linked to care within 1 month of testing and completion of 6- and 12-month visits	Structural	Linkage and retention

Abbreviations: ART, antiretroviral therapy; SMS, short message service.

The first intervention was provision of point-of-care (POC) CD4+ count testing performed immediately after an HIV-positive test, in the same physical location as the HIV testing site, with the aim of improving linkage to care, assessment for ART eligibility, and prompt ART initiation. Several studies have reported higher linkage and ART initiation rates with POC CD4+ count testing as compared to traditional CD4+ count testing [23–26].

The second intervention of accelerated ART initiation for eligible patients (CD4+ count ≤ 350 cells/mm^3^ or WHO stage III/IV) involved 2 rather than 3 counseling sessions and recommended ART initiation within the first week after linkage to care. Delays in ART initiation among those eligible for treatment have been shown to be associated with increased morbidity and mortality [27]. Late ART initiation is also associated with a longer period of increased infectiousness due to ongoing viral replication [28]. In this study, initiation of ART promptly rather than waiting for the return of baseline safety laboratory test results was strongly encouraged, and a checklist was made available to the providers to assist in identifying those potential participants at risk for renal insufficiency who would require waiting for the serum creatinine results prior to ART initiation.

The third intervention involved use of short-message-service (SMS) appointment reminders, sent from a central server, which aimed at improving linkage to and retention in care among participants. SMS reminders were sent 3 days prior to an appointment and after a missed appointment. The message did not contain any information relating to HIV status. SMS communications have been used in HIV care and other chronic disease management to improve health communication and patient adherence [29–37].

The fourth intervention was a health education package that included health information and supplies such as soap, a toothbrush, and a pill box, which also aimed to improve both linkage to and retention in care. A package of different materials and information was given every 3 months. A similar intervention has been evaluated in Uganda and was associated with high rates of cotrimoxazole use, condom use, and HIV testing of family members [38].

Lastly, financial incentives of modest amount were provided that served to reimburse participants for expenses or lost wages or transport costs for clinic attendance [39]. This intervention was selected because there has been great interest in the use of financial incentives as a structural intervention to achieve positive health behaviors including retention in care [39–45]. A noncash type of financial incentive was provided in the form of a prepaid mobile phone card valued at US$8 and was given to participants upon linkage to care within 1 month of HIV testing and at completion of 6 and 12 months in follow-up care.

### Data collection and study measures

All participants completed a baseline questionnaire, at the time of study enrollment, which solicited information on sociodemographic characteristics, HIV disease history, barriers to care, travel time to clinic, depression, social and family support, and HIV-related knowledge. Follow-up questionnaires were conducted at 1 and 12 months after enrollment, at the participant’s home or a prespecified location in the community, to collect information on changes in sociodemographic characteristics, self-reported linkage to care and retention, preferences about the study interventions, and vital status, if the latter was not known. Clinical data including CD4+ count, WHO Stage, date of ART initiation, ART regimen, and clinic and pharmacy visit dates were abstracted from participants’ medical charts—the data source for the primary outcome. These data were collected between 3–6 months after the participant reached the end of the study follow-up period. If the participant’s medical record was missing, he/she was assumed to have not achieved the primary outcome. Death was ascertained via medical record reviews or at the time of the 1- or 12-month interview. Viral load measurement was done using dried blood samples (DBSs) (HIV-1 RNA Abbott m2000rt system) collected at the time of the 12-month questionnaire at the participant’s home or a prespecified location [46].

### Study outcomes

The primary outcome was a combined outcome of linkage to HIV care within 1 month of HIV testing plus retention in care at 12 months from HIV testing among participants at the individual level. Linkage to care was defined by participant attendance of at least 1 visit to an HIV clinic with completion of an intake assessment including medical history and physical exam. Retention in care at 12 months after HIV testing was defined as no documented death and a clinic visit at the study unit within 90 days prior to the end of the study follow-up period. Participants with a missing medical record at the time of medical record abstraction were considered nonretained.

Secondary endpoints included evaluation of the effectiveness of the CIS compared to the SOC with regard to the following: each component of the primary outcome described above, time to linkage, ART eligibility, ART initiation, time to ART initiation, viral suppression defined as HIV-1 RNA < 1,000 copies/mL at 12 months among patients on ART for at least 6 months, and death and loss to follow-up at 12 months after HIV testing. Death and transfer status were ascertained from medical records and through the 12-month follow-up visit questionnaire. Linkage and retention at clinics other than the assigned study unit were assessed in a sensitivity analysis using self-reported linkage and retention data from the 1- and 12-month questionnaires.

### Statistical analysis

The study sample size was calculated by estimating that 35% of the participants in the SOC study arm would achieve the primary outcome (assuming that 50% link to HIV care within 1 month of testing and that 70% of those linking within 1 month are retained at 12 months after testing). We estimated that approximately 2,750 adults would be eligible for study enrollment based on historic HIV testing volume and the proportion of individuals testing HIV positive at the study units in the year prior to the study start. Assuming 80% of eligible individuals would consent to enrollment, we estimated an average enrollment of 220 participants per study unit or 2,200 in total (1,110 per study arm). With this sample size and 5 study units per study arm, we then estimated the minimum difference in the primary outcome we could detect with 80% power, 2-sided alpha of 0.05, assuming an interclass correlation coefficient (ICC) of 0.05. In a post hoc analysis, we estimated the ICC of the primary outcome using the method outlined by Snijders and Bosker for binary outcome data [47].

An intent-to-treat analysis compared the relative risk (RR) of achieving the primary outcome between study arms, with each having 5 clusters per arm. Within study unit clustering was accounted for using random-intercept multilevel models. For dichotomous outcomes, log-Poisson models with robust standard error were used. For continuous outcomes, random-intercept linear regression models were used. Assessment of potential confounding despite cluster randomization was performed by constructing multivariable random-intercept regression models including covariates found statistically different between treatment arms at an alpha of 0.01. Additionally, we conducted a per-protocol analysis comparing the RR of achieving the primary outcome among participants who received the full package of the CIS for the duration of study participation. Sensitivity analysis assessed any changes to the intent-to-treat analysis after including self-reported linkage and retention obtained from follow-up surveys. In post hoc analyses, assessment of the RR for achieving the primary outcome by key subgroups was done using interaction contrast ratios.

## Results

### Study population

**Fig 1:**
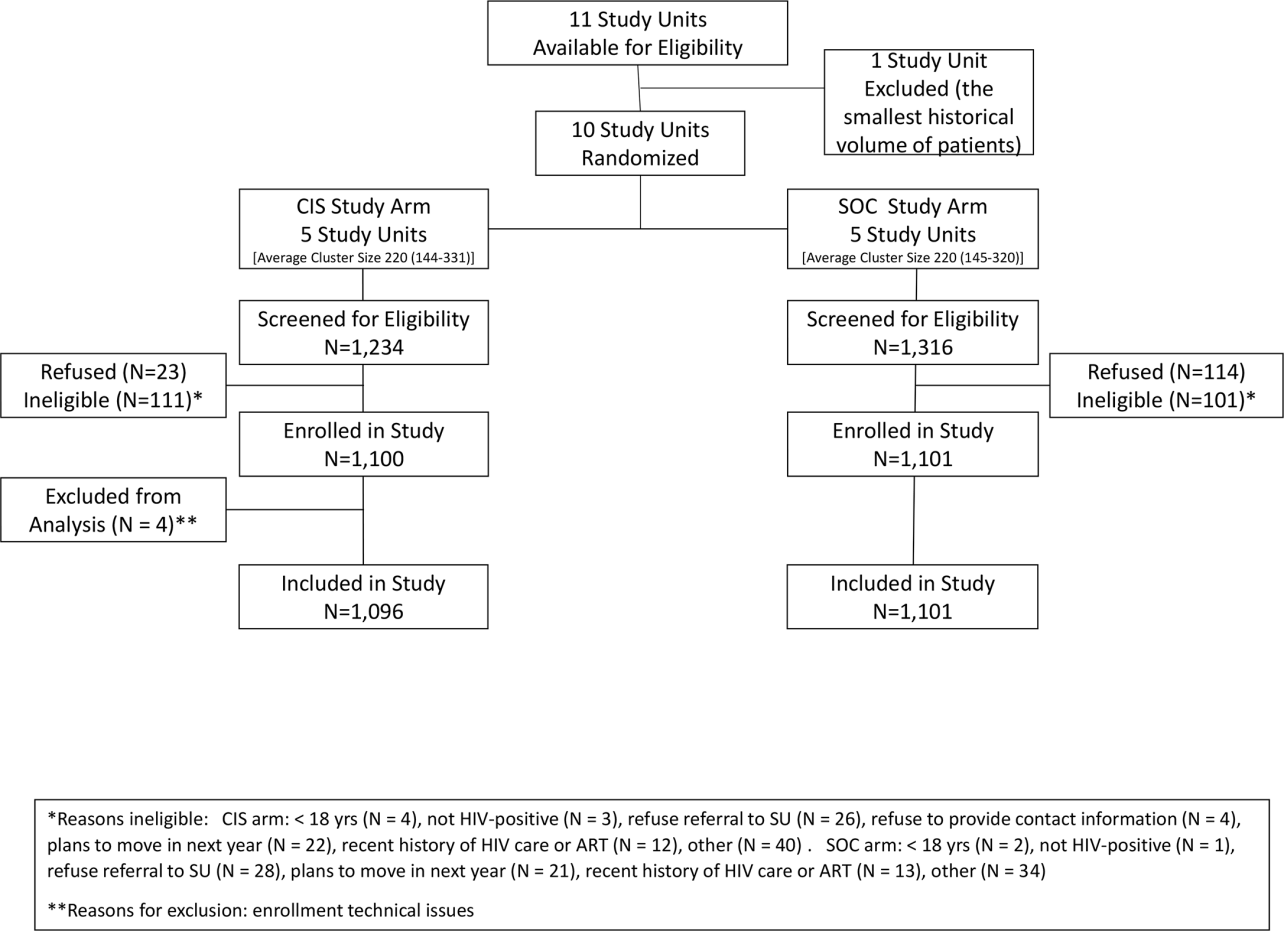
Flow diagram of study enrollment. ART, antiretroviral therapy; CIS, combination intervention strategy; SOC, standard of care; SU, study unit.

Of the 10 study units included in this study, 6 were located in urban areas, and 4 in rural areas. At study units randomized to the CIS study arm, a total of 1,234 individuals were screened for eligibility, with 1,096 (89%) enrolled in the study from 19 August 2013 to 21 November 2014 (Fig 1). At study units assigned to the SOC study arm, a total of 1,316 were screened, with 1,101 (84%) enrolled. Study refusal differed by study arm, with 23 refusals (1.9%) in the CIS arm and 114 refusals (8.7%) in the SOC arm (*p* < 0.001). Reasons for ineligibility are shown in Fig 1, with 111 participants ineligible in the CIS arm compared to 101 in the SOC arm.

Among 2,197 participants included in this analysis, 1,294 (59%) were female, and the median age was 31 years (IQR 26–39), with 445 (20%) of the participants being young adults aged 18–24 years (Table 2). Forty-five percent reported no education or only primary schooling; approximately half were unemployed. Median individual weekly income was US$9 (IQR US$0–US$37). Eighty-four percent reported living in the current residence for more than 1 year, with 16% reporting travel away from home for over a 1-month duration in the past year. The median travel time from residence to HIV clinic was 30 minutes (IQR 20–50). The majority (80%) were diagnosed with HIV through a voluntary counseling and testing site, with the remainder having received HIV testing through provider-initiated testing and counseling at clinics within the study units. Over half (54%) of the participants reported that this was their first HIV test, while 89% indicated that it was their first positive HIV test.

**Table 2:** Participant characteristics at HIV testing (*N* = 2,197).

Characteristics		CIS arm		SOC arm		Total	
		*N*	%	*N*	%	*N*	%
		1,096		1,101		2,197	
**Female**		657	60%	637	58%	1,294	59%
**Age** (years)	Median (IQR)	32 (26–40)		30 (25–39)		31 (26–39)	
	18–24	210	19%	235	21%	445	20%
	25–39	612	56%	604	55%	1,216	55%
	40–49	158	14%	166	15%	324	15%
	>50	116	11%	95	9%	211	10%
	Missing/refused			1	0%	1	0%
**Education**	None/primary	478	44%	519	47%	997	45%
	Secondary or higher	617	56%	581	53%	1,198	55%
	Missing/refused	1	0%	1	0%	2	0%
**Weekly income**	Median (IQR)	US$9 (US$0-US$37)		US$14 (US$0-US$37)		US$9 (US$0-US$37)	
**Unemployed**		624	57%	531	48%	1,155	53%
**Married**		400	36%	408	37%	808	37%
**Number of living children**	0	206	19%	207	19%	413	19%
	1 to 3	645	59%	680	62%	1,325	60%
	>3	243	22%	214	19%	457	21%
	Missing/refused	2	0%	0	0%	2	0%
**Lives alone**		116	11%	160	15%	276	13%
**Away from home >1 month in past year**		179	16%	170	15%	349	16%
**Time at current residence**	1 year or less	164	15%	192	17%	356	16%
	Greater than 1 year	930	85%	906	82%	1,836	84%
	Missing/refused	2	0%	3	0%	5	0%
**Travel time to clinic**	Median (IQR) time minutes	30 (20–45)		30 (20–60)		30 (20–50)	
	<30 minutes	690	63%	584	53%	1,274	58%
	31–60 minutes	330	30%	323	29%	653	30%
	>60 minutes	62	6%	191	17%	253	11%
	Missing/refused	14	1%	3	0%	17	1%
**Currently on TB treatment**		8	1%	14	1%	22	1%
**HIV testing site**	VCT	937	85%	820	74%	1,757	80%
	PITC	159	15%	280	25%	439	20%
	Missing/refused	0	0%	1	0%	1	0%
**First HIV test**		642	59%	539	49%	1,181	54%
**First positive HIV test**		967	88%	978	89%	1,945	89%
**Household member with HIV**		427	39%	348	32%	775	35%
**Alcohol consumption in the last 7 days**	Every day	16	1%	18	2%	34	2%
	Some days	235	21%	234	21%	469	21%
	Never	845	77%	849	77%	1,694	77%

Abbreviations: CIS, combination intervention strategy; PITC, provider-initiated testing and counselling; SOC, standard of care; TB, tuberculosis; VCT, voluntary HIV counselling and testing.

### Primary outcome

**Fig 2:**
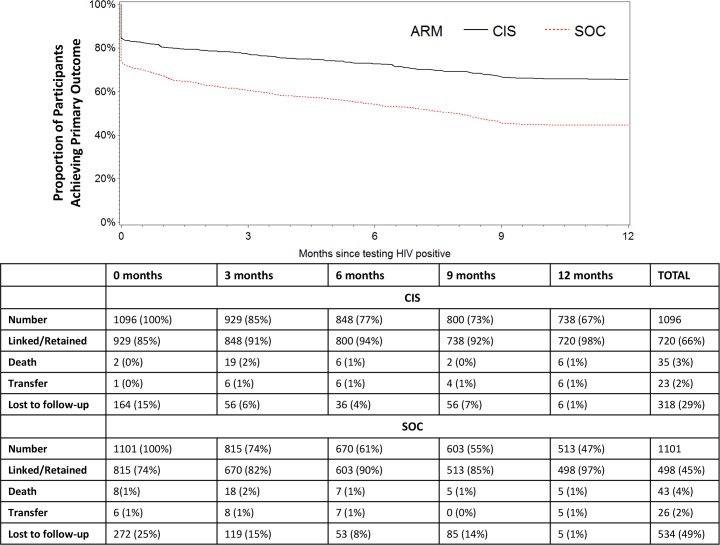
Proportion of participants who achieved the primary outcome of linkage to HIV care within 1 month of HIV testing plus retention in HIV care at 12 months after HIV testing by study arm (combination intervention strategy [CIS] and standard of care [SOC]).

In the intent-to-treat analysis, 705 (64%) participants at sites randomized to the CIS study arm and 477 (43%) participants at sites randomized to the SOC study arm achieved the primary outcome of linkage to HIV care within 1 month of HIV-positive testing plus retention in HIV care at 12 months after HIV testing, for an RR of 1.48 (95% CI 1.37–1.61, *p* < 0.001). Accounting for clustering within study units, the RR was 1.52 (95% CI 1.19–1.96, *p* = 0.002) (Fig 2, Table 3). Additionally, adjusting for covariates significant in the bivariate analyses listed in Table 2 did not appreciably change the results. A total of 64 (6%) of participants in the CIS arm and 144 (13%) of participants in the SOC arm did not have a medical record and were classified as “not linked” to HIV care.

**Table 3:** Primary and secondary outcomes for the combination intervention strategy (CIS) and standard of care (SOC) study arms.

		CIS group (*N* = 1,096)		SOC group (*N* = 1,101)		Relative risk (RR)		
		*N*	%	*N*	%	RR	95% CI	*p*-Value
**Primary outcome**	Intention to treat	705	64%	477	43%	1.48	(1.37–1.61)	<0.001
	Intention to treat accounting for clustering^1^	705	64%	477	43%	1.52	(1.19–1.96)	0.002
	Intention to treat accounting for clustering and differences in covariates^1^^,^^3^	705	64%	477	43%	1.50	(1.12–1.99)	0.009
	Per protocol^1^^,^^2^	672	69%	447	43%	1.68	(1.32–2.15)	<0.001
	Sensitivity analysis^1^^,^^4^	761	69%	557	51%	1.41	(1.13–1.74)	0.004
**Secondary outcomes**								
**Linkage**	Linked to care (ever)^1^	1032	94%	957	87%	1.08	(0.97–1.21)	0.13
	Mean (SD) time from HIV testing to linkage	2.5 days (19.5)		7.5 days (46.6)				0.189
**ART eligibility**	Assessed for ART eligibility^1^	1,096	100%	920	84%	1.20	(1.07–1.34)	0.004
	Became ART eligible^1^	833	76%	721	65%	1.18	(1.01–1.37)	0.038
	Mean (SD) time from HIV testing to ART eligibility assessment^5^	0 (0)		6.3 (35.5)				<0.001
ART initiation*	Initiated ART (ever)^1^*	710	65%	635	58%	1.16	(0.96–1.40)	0.12
	Median (IQR) time from testing HIV positive to ART initiation among ART eligible, days^6^	7.0 (3.0–21.0)		14.0 (7.0–31.0)				<0.001
**Retention regardless of time to linkage and ART status**	Retained 12 months after HIV testing^1^	720	66%	498	45%	1.48	(1.18–1.86)	0.002
**Viral suppression**	Viral suppression (HIV-1 RNA < 1,000 copies/ml) among participants on ART for ≥6 months (*N* = 477 CIS and *N* = 451 SOC)^1^^,^^7^	419	88%	406	90%	0.97	(0.88–1.07)	0.55
**Deaths within 12 months of HIV testing**	Total deaths^1^	35	3%	43	4%	0.80	(0.46–1.35)	0.41
	Death before ART initiation^1^	10	1%	23	2%	0.44	(0.19–1.01)	0.05
	Death after ART initiation^1^	25	2%	20	2%	1.18	(0.57–2.47)	0.63
**Transfers within 12 months of HIV testing**	Total transfers^1^	23	2%	26	2%	0.88	(0.44–1.77)	0.71
	Transfers before ART initiation^1^	7	1%	19	2%	0.37	(0.16–0.85)	0.02
	Transfers after ART initiation^1^	16	1%	7	1%	2.10	(0.72–6.18)	0.16
**Lost to follow-up within 12 months of HIV testing**	Total lost to follow-up^1^	318	29%	534	49%	0.56	(0.40–0.79)	0.002
	Lost to follow-up before ART initiation^1^	240	22%	357	32%	0.60	(0.40–0.89)	0.014
	Lost to follow-up after ART initiation^1^	78	7%	177	16%	0.51	(0.31–0.85)	0.013

^1^ Accounting for within-study unit clustering using random intercept log-Poisson regression models with robust standard error.

^2^ The per-protocol analysis compared all patients in the SOC arm to those in the CIS arm self-reporting receipt of all interventions: point-of-care (POC) CD4+ count, accelerated antiretroviral therapy (ART) initiation (if eligible), health education package, short message service (SMS), and financial incentives. A total of 937 of the 1,096 patients in the CIS arm were included. Patients were excluded for the following: missing PIMA (2), ART counseling session #1 (24), ART counseling session #2 (14), first health education package (7), second health education package (12), third health education package (4), fourth health education package (2), financial incentive for linkage to care (86), second financial incentive (8), or third financial incentive (4).

^3^ Additionally adjusting for covariates significantly different between groups at an alpha of 0.1: employment status, number of children, whether the participant lives alone, HIV testing location, family member with HIV, travel time to clinic, and whether this was the participant’s first HIV test.

^4^ The sensitivity analysis considers participants linked to HIV care or retained in HIV care if they are recorded as linked and retained in their medical records or if they self-reported linkage or retention in the 1- and/or 12-month study questionnaire.

^5^ All participants in the SOC arm were assessed for ART eligibility at the time of testing HIV positive. Of the SOC participants, 920/1,101 (84%) were assessed at enrollment into HIV care or clinical follow-up.

^6^ Time to ART initiation measured from date of HIV-positive test to ART initiation among those becoming ART eligible. The *p*-values are Wilcoxon tests of differences between medians.

^7^ The proportion of viral load suppression (<1,000 copies/ml) among participants who were on ART for ≥6 months with available viral loads is reported in the table. Among all participants who were on ART for ≥6 months, 85% (419/493) in the CIS arm and 89% (406/458) in the SOC arm had viral suppression.

* In the CIS arm, 85% of those ART eligible initiated ART. In the SOC arm, 88% of those eligible initiated ART.

The RR in the per-protocol analysis accounting for clustering for achieving the primary outcome was 1.68 (95% CI 1.32–2.15, *p* = 0.003) (Table 3). The RR in the sensitivity analysis, accounting for clustering, which included participants who self-reported linkage and retention in the 1- and 12-month surveys at a clinic other than 1 with their assigned study unit, was 1.41 (95% CI 1.13–1.74, *p* = 0.004), respectively (Table 3). Using this approach, we calculated an ICC of 0.086, similar to but slightly higher than the assumed ICC used in power and sample size estimation.

The CIS strategy was delivered according to the study protocol to 937 (85%) of the 1,096 participants enrolled in study units assigned to the CIS. Reasons for not receiving all of the CIS strategy intervention components included missing POC CD4+ count testing (<1% CIS participants), missing an ART counseling session per accelerated ART procedures (3%), missing receipt of 1 healthcare bag (2%), and missing receipt of 1 financial incentive (9%). There was heterogeneity in the primary outcome across the 5 pairs of matched study units. The proportion of participants who achieved the primary outcome in study units randomized to the CIS ranged from 49% to 82%, while this ranged from 22% to 57% in the study units randomized to SOC.

### Secondary outcomes

A similar proportion of participants linked to care anytime during the study follow-up period in both study arms: 1,032 (94%) in the CIS arm as compared to 957 (87%) in the SOC arm (RR 1.08, 95% CI 0.97–1.21, *p* = 0.13), with no significant differences in linkage within the same day or 1 month after testing (Table 3). The mean time to linkage to care was shorter in the CIS arm versus the SOC study arm but was not statistically different (2.5 compared to 7.5 days, *p* = 0.189). However, among those who ever linked to care (1,032 in the CIS study arm and 957 in the SOC study arm), significantly fewer patients (13%) in CIS sites versus SOC sites (18%) did not return for subsequent visits after the first clinic visit (*p* = 0.008).

Assessment for ART eligibility through either a CD4+ count or WHO staging was done for all participants in the CIS arm as compared to 84% of participants in the SOC arm (RR 1.20, 95% CI 1.07–1.34, *p* = 0.004). The mean time to ART eligibility assessment was 0 days in the CIS study arm compared to 6.3 days in the SOC arm (*p* < 0.001). The median CD4+ count among 1,096 participants in the CIS arm who had POC CD4+ count testing done at the time of HIV testing was 311 cells/mm^3^ (IQR 159–443). Among the 907 (82%) participants in the SOC arm who linked to HIV care and had a CD4+ count done, the median CD4 count was 285 cells/mm^3^ (155–444) (*p* = 0.07).

A total of 710 participants (85% of ART-eligible participants) in the CIS arm as compared to 635 (88% among ART-eligible participants) in the SOC arm initiated ART within the study follow-up period (RR 1.16, 95% CI 0.96–1.40, *p* = 0.12) (Table 3). The median time from HIV testing to ART initiation among eligible patients was 7.0 days (IQR 3.0–21.0) as compared to 14.0 days (IQR 7.0–13.0) in the CIS and SOC study arms, respectively (*p* < 0.001).

Retention in care, regardless of time to linkage or ART status, at 12 months was significantly greater in participants in the CIS as compared to the SOC study arm, with an RR of 1.48 (95% CI 1.18–1.86, *p* = 0.002). Loss to follow-up during pre-ART care (RR = 0.60, 95% CI 0.40–0.89, *p* = 0.014) and after ART initiation (RR = 0.51, 95% CI 0.31–0.85, *p* = 0.013) was significantly lower in the CIS arm as compared to the SOC arm.

For participants on ART for at least 6 months during follow-up regardless of retention status, viral load data were available for 97% (*N* = 477/493) of participants in the CIS arm and 98% (*N* = 451/458) in the SOC arm. Viral suppression among participants on ART ≥6 months with available viral loads was similar by study arm at 88% in CIS and 90% in SOC (RR 0.97, 95% CI 0.88–1.07, *p* = 0.55).

**Fig 3:**
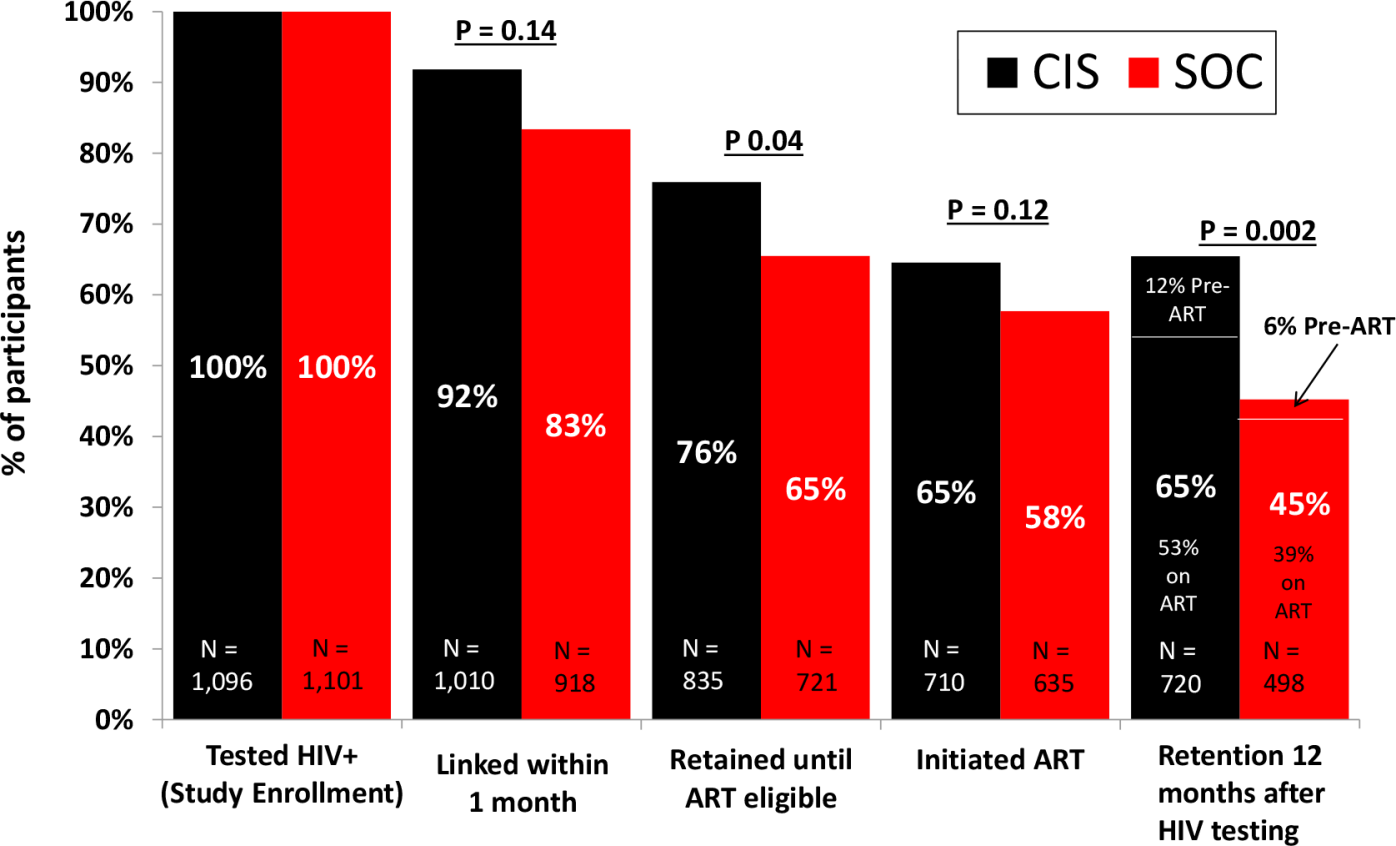
HIV care continuum comparing the combination intervention strategy (CIS) study arm versus the standard of care (SOC) study arm.

There were 78 deaths (3.6% of the study population) that occurred during follow-up, and this did not differ by study arm (35 deaths [3%] in the CIS study arm versus 43 deaths [4%] in the SOC arm, with an RR of 0.80, 95% CI 0.46–1.35, *p* = 0.40) (Table 3). However, there was nonsignificantly lower mortality among participants prior to ART initiation in the CIS arm (10 deaths) compared to the SOC arm (23 deaths), with an RR of 0.44 (95% CI 0.19–1.01, *p* = 0.05). Fig 3 compares the CIS study arm versus the SOC study arm across the HIV care continuum from linkage to care within 1 month of testing through retention in care at 12 months after testing HIV positive.

**Fig 4:**
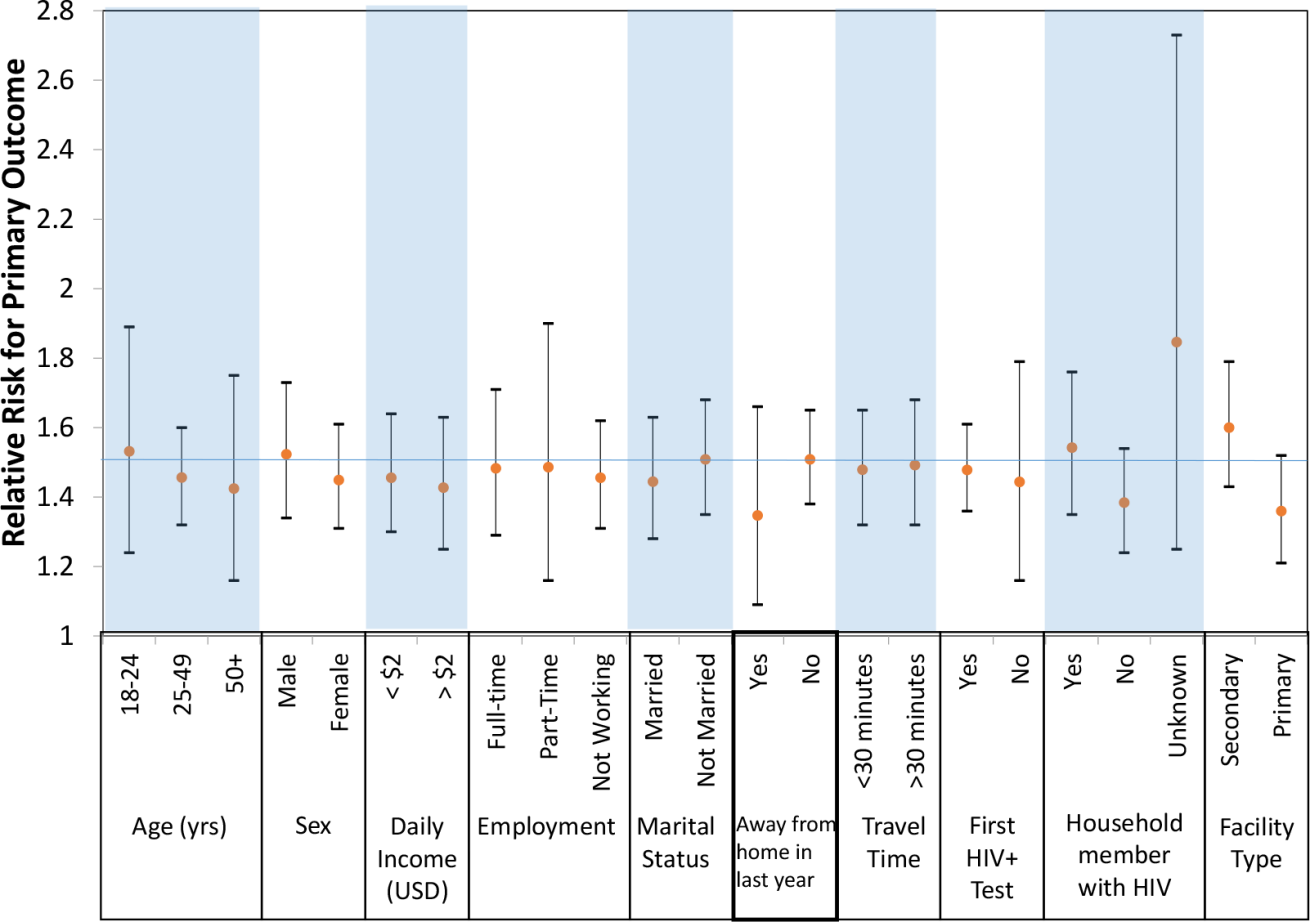
Primary outcome by subgroups of participants. USD, US dollars; yrs, years.

In post hoc analyses, we examined achievement of the primary outcome between study arms by key subgroups. The effect of the CIS, as compared to the SOC, was consistent across all prespecified subgroups, including by age, sex, income, employment, marital status, travel away from home in the past year, travel time to clinic, past HIV testing history, household members with HIV, and type of clinic (Fig 4, S1 Table).

## Discussion

In this cluster-randomized study, a novel combination strategy, inclusive of 5 evidence-based interventions, was 50% more effective than the SOC in enhancing linkage to care plus retention in care among HIV-positive individuals. The robustness of this outcome is supported by the consistent findings in the per-protocol analysis, in sensitivity analyses, and across subgroups of participants. In addition, the combination strategy was associated with improvements across multiple steps of the care continuum, with an increased proportion of participants who were assessed for ART eligibility, decreased time to ART eligibility assessment, decreased time to ART initiation, increased retention at 12 months after HIV testing regardless of time to linkage and ART status, and decreased mortality among participants prior to ART initiation. However, high rates of viral suppression were similar among ART patients by study arm.

In our study, the effect noted on the primary outcome appeared to be largely driven by enhanced retention rather than by the linkage-to-care component. This finding may be due to the high proportion of participants in both study arms who linked to care within 1 month of HIV testing in both arms of the study (87% in the SOC arm and 92% in the CIS arm), and thus, our sample size was insufficient to show a difference between the arms. The high proportion of participant linkage was likely influenced by a national campaign to improve linkage that was implemented during the study period [21].

The combination strategy significantly reduced loss to follow-up among participants regardless of whether they were in pre-ART care or on treatment. Loss to follow-up, in both study arms, was higher among pre-ART participants, as compared to participants who had initiated ART. This is consistent with findings from other studies, including those from a large study of 390,603 HIV-positive adults in Kenya, Mozambique, Rwanda, and Tanzania, in which 34.8% of all patients who had not initiated ART were lost from care at 12 months, compared to 5.8% among patients on ART [6]. While the pre-ART care phase should largely be minimized with the release of the recent WHO guidelines that recommend offering ART to all HIV-positive individuals irrespective of CD4+ count or WHO disease stage, evidence suggests that retention in care and on ART remains a challenge even in the context of “treatment for all” [48]. For example, while adoption of Option B+, which entails initiation of ART for all HIV-positive pregnant women, has been associated with an increase in the number of pregnant women on ART, loss to follow-up has remained a challenge. Among 21,939 HIV-infected pregnant women who started ART as per Option B+ in Malawi, 17% were lost to follow-up at 6 months after treatment start, with a 5-fold higher loss to follow-up compared to those who initiated ART at a more advanced stage of HIV disease [49]. Thus, the findings from our study remain relevant even though the study was conducted at a time when a CD4+ count threshold was recommended for ART initiation.

In this study, viral suppression was high among all participants on ART for a minimum of 6 months, irrespective of study arm. This confirms the potency of the first-line regimen, consisting of tenofovir, lamivudine, and efavirenz or nevirapine, and suggests that participants were highly adherent to their medications. These findings build upon those from the Population-based HIV Impact Assessment Project surveys that were conducted recently in Malawi, Zambia, Zimbabwe, and Swaziland, which included nationally representative samples of individuals in which 87% of HIV-positive adults who reported being on ART were virally suppressed (HIV-1 RNA < 1,000 copies/ml) [50,51]. Findings from this project survey in Swaziland showed that among adults who were aware of their HIV-positive status and who indicated being on ART, 92% had a suppressed viral load [52]. The finding of similarly high proportions of viral suppression among participants in both arms of our study suggests that the sample size was insufficient to detect a difference. In addition, it is important to note that the interventions used in this study were not designed with a focus specifically on enhancing medication adherence and viral suppression. Design of future combination strategies may prioritize the use of interventions that focus specifically on enhancing adherence to ART, such as the use of financial incentives to improve viral suppression [53].

Every effort was made to ascertain accurate loss to follow-up and mortality outcomes in our study. It should be noted that reporting of accurate loss to follow-up and mortality outcomes by HIV programs has been a controversial topic. This is due to the fact that when tracing was done for individuals reported as lost to follow-up by HIV programs, a substantial proportion were found to have either died or transferred care to another health facility [54]. We feel confident that it is unlikely that such misclassification occurred in our study as home tracing was conducted for all study participants to ascertain their outcomes at the end of the 12-month follow-up period. While the study was not powered to detect a difference between the study arms in terms of mortality, the combination strategy appeared to have a meaningful—albeit not statistically significant—effect, with as much as 50% lower mortality noted among pre-ART patients. This may be due to better retention in care among participants in the intervention arm. Poor retention in care has been demonstrated to be associated with increased mortality, likely due to missed clinic visits that deprive patients of clinical and laboratory assessments for diagnosis of early complications and delay prompt initiation of ART [55].

We observed substantial heterogeneity in the primary outcome across clinics in both the CIS and SOC study arms. This may reflect clinic-level differences such as clinic size and location. For example, the CIS study unit with the lowest achievement of the study’s primary outcome was the largest clinic in urban Swaziland. It is possible that individuals who test HIV positive at such a large clinic may seek ongoing care at clinics closer to their homes. Other reasons could be differences in patient-level factors, such as sex, age, and immunological status, which warrant further analyses.

To date, most intervention studies to address gaps in the HIV care continuum have focused on 1 step in the continuum, largely that of ART initiation. The Rapid Initiation of Treatment trial showed that single-visit ART initiation that included POC CD4+ count testing was associated with significantly higher ART initiation (97%) compared to the standard of care (72%) [56]. The START-ART trial was a stepped-wedge cluster-randomized trial of 20 clinics in Uganda that evaluated an intervention aimed at improving ART initiation among eligible patients; this intervention was associated with a higher proportion of patients initiating ART (80%) within 14 days after determination of ART eligibility compared to 38% in the control group [13]. Finally, the Same Day ART Initiation Study in Haiti, which evaluated the effect of same-day ART initiation on the day of HIV diagnosis among asymptomatic HIV-positive adults with CD4+ count ≤ 500 cells/uL and WHO stage I or II disease, noted that a higher proportion (53%) of participants randomized to same-day ART initiation were retained in care at 12 months with viral suppression compared to those in the standard of care arm (44%) [14].

Our study had several strengths, including the use of a pragmatic approach consistent with implementation science design. Specifically, it utilized broad eligibility criteria, was conducted within established health facilities, tested feasible interventions that were delivered primarily by available staff rather than research staff, and assessed the primary outcome largely through routinely available data. In addition, the study included the majority of clinics in Swaziland and involved cluster-randomized design rather than randomization of individual participants, which allowed for ease of implementation and avoided disruption of services within the clinics. The study also uniquely assessed the effect of the delivery of multiple interventions packaged in 1 strategy aimed at multiple steps in the HIV care continuum. Thus, implementation of the study strategy has the potential to achieve not only prompt ART initiation but also better retention in care and on ART, consequently enhancing individual and society benefits from the “treat all” approach.

The primary limitations of this study included a limited number of clusters, although it was inclusive of all the available clusters in the country. At the time of study initiation, there were only 11 secondary health facilities offering HIV services in Swaziland, and we selected 10 of these for participation in this study. Consequently, it is possible that the cluster randomization did not evenly distribute all determinants of linkage and retention other than the study interventions between treatment arms. While it is encouraging that analyses adjusting for individual-level differences between treatment arms did not appreciably change the results, we cannot definitively rule out residual confounding as a potential explanation of the findings. In addition, the design focused on evaluation of a package of interventions as 1 strategy and, thus, it did not allow for evaluation of the effectiveness of individual components of the combination approach. Another limitation was use of self-reported linkage and retention at other clinics to ascertain undocumented transfers to other clinics outside of the study unit.

### Conclusions

The Link4Health study demonstrated that a combination strategy of evidence-based interventions, aimed at gaps in various steps of the HIV care continuum, was highly effective in enhancing linkage of HIV-positive individuals to care plus increasing their retention in care and on ART. The study also showed that once participants initiated ART, viral load suppression was high irrespective of the study arm. Cost effectiveness and qualitative analyses are ongoing in order to inform decision makers considering adoption of this strategy. Our findings offer an effective strategy that can advance the quality of HIV programs in Swaziland and that can be adapted to other similar contexts.

## Supporting information

S1 Text Consolidated Standards of Reporting Trials (CONSORT) statement. (DOCX)

S1 Data Link4Health deidentified dataset. (XLSX)

S1 Table Primary outcome by prespecified participant subgroup. (DOCX)

## Abbreviations

ART: antiretroviral therapy
CIS: combination intervention strategy
CONSORT: Consolidated Standards of Reporting Trials
DBS: dried blood sample
ICC: interclass correlation coefficient
PITC: provider-initiated testing and counselling
POC: point of care
RR: relative risk
SMS: short message service
SOC: standard of care
SU: study unit
TB: tuberculosis
UNAIDS: Joint United Nations Programme on HIV/AIDS
VCT: voluntary HIV counselling and testing
