# Supplementary material for: Effectiveness of a combination strategy for linkage and retention in adult HIV care in Swaziland: The Link4Health cluster randomized trial
Source: PLoS Med. 2017 Nov 7;14(11):e1002420. doi: 10.1371/journal.pmed.1002420 (PMC5675376; doi:10.1371/journal.pmed.1002420)
Supplement: S1 Table — (DOCX) [file pmed.1002420.s003.docx]

**S1 Table: Primary outcome by pre-specified participant subgroup**

|  | | **CIS Study Arm** | | **SOC Study Arm** | | **Relative Risk** | **95% CI** | **Interaction Contrast Ratio** | **P-value** |
| --- | --- | --- | --- | --- | --- | --- | --- | --- | --- |
|  |  | N | % | N | % |  |  |  |  |
| **Age** | 18-24 | 115 | 55% | 84 | 36% | 1.53 | (1.24-1.89) | -1.4% | 0.97 |
|  | 25-49 | 498 | 65% | 341 | 44% | 1.46 | (1.33-1.61) | Reference | |
|  | >50 | 92 | 79% | 52 | 55% | 1.42 | (1.18-1.78) | 4.2% |  |
| **Sex** | Male | 285 | 64% | 196 | 42% | 1.52 | (1.34-1.73) | 1.9% | 0.52 |
|  | Female | 420 | 64% | 281 | 44% | 1.46 | (1.31-1.62) | Reference | |
| **Income** | < $2per day | 322 | 65% | 227 | 45% | 1.46 | (1.30-1.64) | 2.2% | 0.60 |
|  | > $2 per day | 224 | 61% | 205 | 42% | 1.44 | (1.26-1.64) | Reference |  |
| **Employment** | Full-time | 195 | 62% | 184 | 41% | 1.49 | (1.29-1.71) | -0.9% | 0.91 |
|  | Part-time | 94 | 61% | 51 | 41% | 1.49 | (1.16-1.90) | -1.2% |  |
|  | Unemployed | 416 | 67% | 242 | 46% | 1.46 | (1.31-1.63) | Reference |  |
| **Marital status** | Married | 279 | 70% | 197 | 48% | 1.44 | (1.28-1.63) | 0.6% | 0.81 |
|  | Not Married | 424 | 61% | 279 | 40% | 1.52 | (1.36-1.69) | Reference |  |
| **Away from home > 1 month over past year** | Yes | 107 | 60% | 75 | 44% | 1.35 | (1.10-1.67) | -6.5% | 0.70 |
|  | No | 598 | 65% | 399 | 43% | 1.51 | (1.39-1.65) | Reference |  |
| **Travel time to clinic** | < 30 minutes | 435 | 63% | 249 | 43% | 1.48 | (1.36-1.65) | 0.03% | 0.59 |
|  | > 30 minutes | 261 | 64% | 227 | 44% | 1.51 | (1.34-1.70) | Reference |  |
| **First positive HIV test** | Yes | 615 | 64% | 419 | 43% | 1.48 | (1.36-1.62) | -0.68% | 0.98 |
|  | No | 90 | 70% | 58 | 48% | 1.44 | (1.16-1.79) | Reference |  |
| **Household member with HIV** | Yes | 299 | 70% | 158 | 45% | 1.54 | (1.35-1.76) | 7.4% | 0.77 |
|  | No | 374 | 61% | 293 | 44% | 1.39 | (1.25-1.55) | Reference |  |
|  | Missing | 32 | 57% | 26 | 31% | 1.85 | (1.25-2.73) | 9.0% |  |
| **Clinic Type** | Secondary | 452 | 61% | 262 | 38% | 1.61 | (1.44-1.80) | 3.9% | 0.29 |
|  | Primary | 253 | 72% | 215 | 53% | 1.36 | (1.22-1.53) | Reference |  |
